# Hypermethylation of *DHRS3* as a Novel Tumor Suppressor Involved in Tumor Growth and Prognosis in Gastric Cancer

**DOI:** 10.3389/fcell.2021.624871

**Published:** 2021-01-21

**Authors:** Sha Sumei, Kong Xiangyun, Chen Fenrong, Sun Xueguang, Hu Sijun, Bai Bin, Shi Xiaolei, Tu Yongjiu, Wu Kaichun, Zhao Qingchuan, Nie Yongzhan, Xu Bin

**Affiliations:** ^1^The Second Affiliated Hospital of Xi’an Jiaotong University, Xi’an, China; ^2^State Key Laboratory of Cancer Biology, Xijing Hospital of Digestive Diseases of the Air Force Medical University, Xi’an, China; ^3^Xi’an No.1 Hospital, Xi’an, China; ^4^Division of Human Genetics, Cincinnati Children’s Hospital Medical Center, Cincinnati, OH, United States; ^5^Dongfang Hospital of Xiamen University, Fuzhou, China; ^6^The General Surgery Department of Chenggong Hospital of Xiamen University (Central Hospital of the 73th Chinese People’s Liberation Army), Xiamen, China

**Keywords:** gastric cancer, DNA methylation, *DHRS3*, Mass-Array, carcinogenesis

## Abstract

**Background/Aims:**

The role of DHRS3 in human cancer remains unclear. Our study explored the role of *DHRS3* in gastric cancer (GC) and its clinicopathological significance and associated mechanisms.

**Materials:**

Bisulfite-assisted genomic sequencing PCR and a Mass-Array system were used to evaluate and quantify the methylation levels of the promoter. The expression levels and biological function of *DHRS3* was examined by both *in vitro* and *in vivo* assays. A two-way hierarchical cluster analysis was used to classify the methylation profiles, and the correlation between the methylation status of the *DHRS3* promoter and the clinicopathological characteristics of GC were then assessed.

**Results:**

The *DHRS3* promoter was hypermethylated in GC samples, while the mRNA and protein levels of *DHRS3* were significantly downregulated. Ectopic expression of *DHRS3* in GC cells inhibited cell proliferation and migration *in vitro*, decreased tumor growth *in vivo*. *DHRS3* methylation was correlated with histological type and poor differentiation of tumors. GC patients with high degrees of CpG 9.10 methylation had shorter survival times than those with lower methylation.

**Conclusion:**

*DHRS3* was hypermethylated and downregulated in GC patients. Reduced expression of *DHRS3* is implicated in gastric carcinogenesis, which suggests *DHRS3* is a tumor suppressor.

## Introduction

The methylation patterns of DNA are highly variable among cell types and developmental stages and are influenced by environment and lifestyle (Verma and Manne, 2006; Horvath, 2013). DNA methylation is mainly found at the 5′-position of cytosine residues (5 mC or 5-methyl cytosine) followed by a guanine dinucleotide sequence (CpG). Regions in the genome that are characterized by a particularly high CpG content are termed “CpG islands,” and are present in approximately 60% of human gene promoters (Portela and Esteller, 2010). Aberrant DNA methylation has been linked to a variety of common diseases, especially cancer (Bock, 2009; Zhang and Jeltsch, 2010; Klutstein et al., 2016). It is now known that DNA methylation often occurs in gene promoters and is associated with transcriptional silencing of tumor suppressors or other genes important for normal cellular function (Corvalan, 2013; Huang et al., 2015; Gyorffy et al., 2016). Moreover, these alterations are usually among the earliest and most frequent molecular events known to occur during tumorigenesis (Akhavan-Niaki and Samadani, 2013). Great efforts have been made to identify novel methylation-based markers that can be used not only for the early detection of cancer, but also for determining risk, monitoring tumor progression, evaluating therapy response, and potentially for designing target specific epigenetic drugs (Beltran et al., 2008; Madu and Lu, 2010; Marzese and Hoon, 2015). Gastric cancer (GC) is the fifth most common cancer and the third leading cause of cancer-related mortality worldwide (Torre et al., 2012). During the past few decades, extensive studies have been carried out to understand the molecular biology of GC. However, its molecular mechanisms are still not fully understood. Genetic and epigenetic alterations of tumor related genes have been studied widely (Tahara and Arisawa, 2015; Yoda et al., 2015). It was recently reported that the methylation of some molecules, such as cadherin 4, protocadherin 10, and Runt-related transcription factor 3 (Satoh et al., 2004; Yu et al., 2009; Llorca-Cardenosa et al., 2016), predisposes patients to GC, suggesting that DNA methylation may play an important role in GC tumorigenesis.

The human short-chain dehydrogenase/reductase (SDR) protein gene *DHRS3* was first identified in 1998 (Haeseleer et al., 1998), and is ubiquitously expressed in tissues and catalyzes the oxidation/reduction of a wide range of substrates including retinoids and steroids (Lundova et al., 2015). Deletion of *DHRS3* results in loss of local retinol storage, leading to shortage of vitamin A active metabolites and thus contributing to cancer development and progression (Cerignoli et al., 2002). Some studies have found monoallelic deletion of *DHRS3* in some neuroblastoma cell lines, but others have shown that *DHRS3* is constitutively expressed in breast cancer cell lines and highly expressed in papillary thyroid carcinoma (Haeseleer et al., 1998; Oler et al., 2008). Nevertheless, the differential expression patterns of *DHRS3* in different tumors indicate the context-dependent functions of this gene. Further study is needed to clarify the actual physiological roles of *DHRS3* in tumorigenesis in different types of tumors.

Recently, we utilized *in silico* sequence analysis to identify a long CpG island with high CpG density in the 5′ leader region of the *DHRS3* gene, spanning the gene promoter and the first exon. Here, we further evaluated the epigenetic regulation, expression profile, biological function, and clinical association of *DHRS3* in GC.

## Materials and Methods

### Clinical Samples and Cell Lines

To detect methylation, paired primary cancer tissues and corresponding non-tumor tissues were obtained from 60 patients [10 for bisulfite-assisted genomic sequencing PCR (BSP), 50 for Mass-Array] with primal GC who had not received chemotherapy or radiotherapy before the operation. Clinical and pathological data were obtained from the surgical pathology records. The tissues were frozen in liquid nitrogen immediately after resection, and stored at −80°C. All patients provided informed consent before collection of the specimens in accordance with the guidelines of our institution. Our institution preserved four GC cell lines (SGC7901, MKN28, MKN45, and AGS) and one gastric epithelial cell line (GES). MKN28 cells were transfected with the *DHRS3* gene or empty vector using a lentiviral expression system (Genechem, Shanghai, China), cultured in 6-well plates, and continuously selected with puromycin to generate stable cell lines. The expression of *DHRS3* in cultured cells was confirmed by western blot via an anti-*DHRS3* antibody. Cells were cultured in RPMI-1640 medium supplemented with 10% fetal bovine serum (FBS), 80 units/mL penicillin and 100 mg/mL streptomycin at 37°C under 5% CO_2_.

### DNA Extraction and Bisulfite Modification

Genomic DNA was extracted from gastric tissues and cell lines using a QIAamp Mini kit (QIAGEN, Hilden, Germany) according to the manufacturer’s instructions, and analyzed by electrophoresis on 1.0% Tris/Borate/EDTA agarose gel containing 1% ethidium bromide. The DNA samples were modified by sodium bisulfite using a Zymo DNA Methylation-Gold kit (Zymo Research, Orange, CA) according to the manufacturer’s instructions.

### Bisulfite-Assisted Genomic Sequencing Analysis

Ten paired tissue specimens (also used for quantitative real-time PCR) were used for BSP. Briefly, genomic DNA treated with 2.5 μL of bisulfite was used as the template for amplification. The primer for the *DHRS3* promoter was designed using a web-based program (MethPrimer^1^) by the input of a 1 kb sequence upstream of the transcription start site (TSS) of *DHRS3* (Li and Dahiya, 2002); the following primer was used: *DHRS3* BSP: TTTTGTTTTTTTTAATTTGGAGAGG, ATCAAACTTTTAAAAATCCACTCTAC, 623 bp. The PCR products were cloned into a pMD 18-T Vector (TaKaRa, Dalian, China). Six clones were randomly chosen, and plasmids were extracted using a TIANprep Mini Plasmid Kit (TIANGEN, Beijing, China) and sequenced using an ABI 3730 analyzer (Applied Biosystems, Foster City, CA). The most representative sequence for each sample was selected for analysis using a BiQ analyzer (Bock et al., 2005).

### Mass-Array Analysis

To quantitatively determine the methylation status of the CpG islands of *DHRS3* in 50 paired tumor samples, the high-throughput quantitative methylation analysis platform Mass-Array (Sequenom, San Diego, CA) was carried out as described previously (Radpour et al., 2008, 2009). This system uses matrix-assisted laser desorption/ionization time-of-flight mass spectrometry in combination with RNA base-specific cleavage. Briefly, a bisulfite-treated template was amplified using the same primers as those for bisulfite sequencing. The products were purified and then spotted on a 384-pad SpectroCHIP, followed by spectral acquisition on a Mass-Array Compact System. These signal pairs were used to estimate the ratio of methylated to unmethylated DNA. Methylation data of individual units (1–5 CpG sites per unit) were generated and analyzed using EpiTyper software (Agena Bioscience, Inc., San Diego, CA).

### Demethylation With the DNA Demethylating Agent 5-Aza-2′-Deoxycytidine in GC Cells

SGC-7901, MKN28, MKN45, and AGS cells at 20% confluence were treated with 2 μmol/L of the DNA demethylating agent 5-Aza-2′-deoxycytidine (5-Aza, Sigma-Aldrich, St. Louis, MO) for 72 h. Cells were then harvested for RNA extractions.

### Reverse-Transcription PCR and Quantitative Real-Time PCR

Total RNAs were extracted with Trizol reagent (Invitrogen, Carlsbad, CA). The mRNA expression level of *DHRS3* was measured by quantitative real-time PCR, using the following primer: *DHRS3*: CATGGGAAGAGCCTAATGGA, GACGCTTTGGATGTGCAGTA; 200 bp. The program was performed using a Light-Cycler 480 system (Roche Diagnostics, Risch-Rotkreuz, Switzerland) with the SYBR Premix Ex Taq II (TaKaRa) reagent. Amplification of each sample was repeated three times, and the specific product was analyzed by melting curve at the end of the program. The housekeeping gene glyceraldehyde-3-phosphate dehydrogenase was used as an internal control. Gene expression level was quantified by the comparative CT method.

### Immunohistochemical Staining

Immunohistochemical staining of *DHRS3* was performed on parallel histopathological sections from paraffin-embedded tumor sections and corresponding adjacent normal tissue using an anti-*DHRS3* antibody (Biosynthesis Biotechnology, Beijing, China) diluted at 1:300. All sections were examined microscopically and scored by three independent pathologists blinded to the clinical and pathological information. The expression of *DHRS3* was evaluated according to the ratio of positive cells per specimen and staining intensity. The ratio of positive cells per specimen was evaluated quantitatively and scored 0 for staining of 0–1%, 1 for staining of 2–25%, 2 for staining of 26–50%, 3 for staining of 51–75%, and 4 for staining >75% of the cells examined. Intensity was graded as follows: 0, no signal; 1, weak; 2, moderate; and 3, strong staining. A total score of 0–12 was calculated using the following formula: total score = ratio of positively stained cells (score) × intensity of immunoreactivity (score) and graded as negative (I; score: 0–1), weak (II; 2–4), moderate (III; 5–8), or strong (IV; 9–12).

### Cell Proliferation Assay

Stable transfected cells were seeded in 96-well plates at a density of 3,000 cells per well and grown under normal conditions. Viable cell counts were determined in triplicate on days 1–7 using the MTT assay.

### Colony Formation Assay

Cells were infected with *DHRS3* or the empty vector using a lentiviral expression system and plated in 6-well plates at 300 cells/well. After 2 weeks, colonies stained with Giemsa solution were counted. The assay was performed in triplicate.

### Wound-Healing Assays

Cells were seeded into 24-well plates and cultured until 80–90% confluence. A 10-μL pipette tip was used to make a vertical wound. Then, cells were washed three times with culture medium to remove cell debris. The extent of wound closure was monitored at designated time points in the same position.

### Transwell *in vitro* Migration Assays

Cells in serum-free medium (200 μL containing 5 × 10^4^ cells) were added to the top chambers of transwell chambers. The bottom chamber contained medium with 20% FBS as a chemoattractant. The cells were incubated for 24 h at 37°C. Then, cells that had migrated through the membrane and attached to the lower surface were stained using a fixative/staining solution (0.1% crystal violet, 1% formalin, and 20% ethanol) and quantified under a microscope. The tests were repeated three times.

### Flow Cytometry Analysis

MKN28 cells were infected with *DHRS3* or empty vector using a lentiviral expression system. Cells were harvested and fixed in 70% ice-cold ethanol for 1 h, washed with phosphate-buffered saline (PBS), and stained with propidium iodide (PI) in PBS supplemented with RNase for 20 min. Cells were sorted by FACScan (BD Biosciences, Franklin Lakes, NJ). Cell apoptosis assays were performed using the annexin V/PI kit by flow cytometry analysis. The stained cells were finally analyzed by FACScan.

### Tumorigenicity Assay in Nude Mice

MKN28 cells infected with *DHRS3* or control lentivirus were injected subcutaneously into the lateral root of one posterior limb of nude mice (2 × 10^6^ cells/mouse; five mice in each experimental group). Tumor length was measured every week for 5 weeks. The care of the experimental animals was in accordance with the institutional animal care and use committee guidelines.

### Statistical Analysis

Data analysis was performed using SPSS version 17.0 (Chicago, IL). The Shapiro-Wilk test was used to analyze normality. The mRNA expression dataset was not normally distributed. The Wilcoxon signed ranks test was used to analyze the difference in expression levels between paired tumor specimens and the corresponding adjacent non-tumor tissue specimens. Independent-samples *t*-test was used for two groups of data and one-way analysis of variance was used for three groups of data to compare methylation levels between the sample groups. Using two-way hierarchical cluster analysis, we examined the relationships between methylation levels in CpG sites and tissues (Radpour et al., 2008). Overall survival in relation to methylation status was evaluated using the Kaplan-Meier survival curve. Two-sided values of *P* < 0.05 were considered statistically significant.

## Results

### *DHRS3* Promoter Was Hypermethylated in GC Patients

**FIGURE 1:**
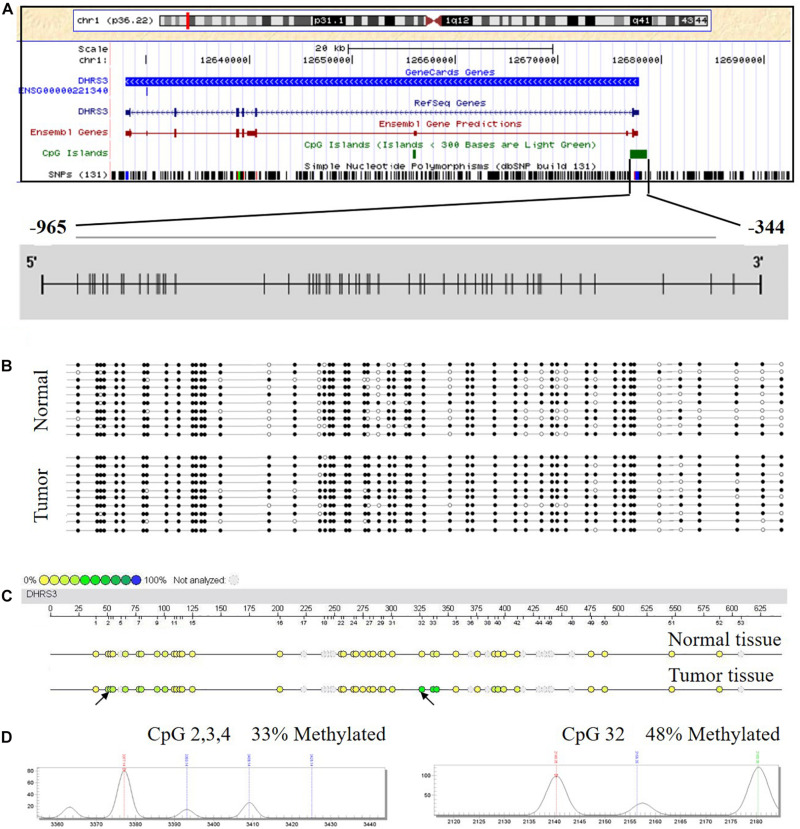
The DHRS3 promoter in human GC tumors and cell lines is hypermethylated. **(A)** Gene location, amplicon, and CpG sites (short vertical lines) in relation to the promoter of DHRS3. **(B)** A simplified illustration of BSP reads for a tumor sample and its adjacent normal sample. Open and filled circles represent unmethylated and methylated CpG sites, respectively. **(C)** Methylation profile of CpG sites of the DHRS3 gene. The colors of the circles are related to the percentage methylated in each CpG site. Arrows indicate different methylation patterns between cancerous tissues and adjacent paired normal tissues. **(D)** Double dendrogram of the DHRS3 gene. Peaks show the methylation rates of the two CpG sites in the DHRS3 gene, which indicate differences between cancerous and paired normal tissues.

Using *in silico* sequence analysis, we identified a long CpG island with high CpG density in the 5′ leader region of *DHRS3* gene that spanned the gene promoter and the first exon, implying the potential for epigenetic regulation via DNA methylation. To test this hypothesis, we examined the methylation status of *DHRS3* promoters in 10 human GC tissues and 10 corresponding normal mucosa samples by BSP. The region we examined started from 344 bp upstream of the TSS, extended for another 621 nt upward, and contained 53 CpGs (Figure 1A). We found different methylation patterns of the *DHRS3* promoter between the tumor and normal tissues. *DHRS3* promoters in tumor samples were generally hypermethylated (Figure 1B). Moreover, the differently methylated regions were unevenly distributed along the promoter, with some sites more pronounced than others, such as CpG 16, 18, 25, 26, 32, 37, 38, 40, 41, 48, and 52. These results indicated the presence of primary sites of methylation, which may be critical for the maintenance of gene repression, and candidate sites for the initiation of inactivation (Park and Chapman, 1994).

### Quantitative Methylation Analysis and Identification of Hypermethylated CpG Sites

**FIGURE 2:**
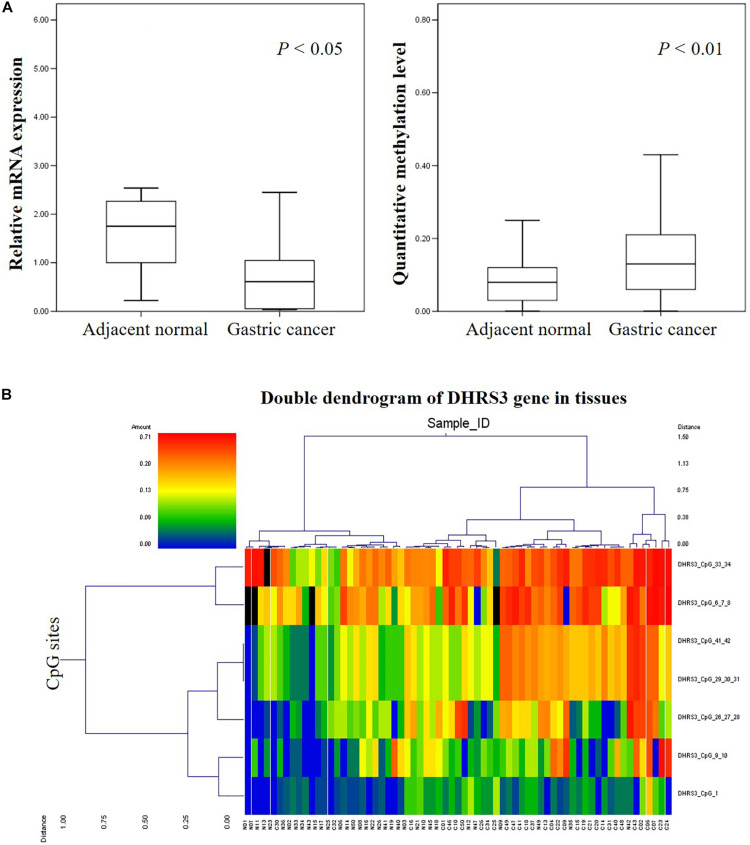
High-throughput analysis of the informative CpG sites for the DHRS3 gene. **(A)** DHRS3 was hypermethylated and lacked mRNA expression in GC. (1) Box plots indicating the relative expression levels of DHRS3 mRNA in 21 paired GC tissues. Sample one served as a calibrator. *P*-values were estimated using a paired two-sample *t*-test. (2) Box plots indicating the quantitative DNA methylation analysis by Mass-Array. *P*-values were estimated using an independent samples *t*-test. **(B)** Two-way hierarchical cluster analysis of tumor tissues and adjacent normal tissues (red clusters are 71% methylated, green clusters are 0% methylated). The color gradient between red and green indicates methylation ranging from 0 to 71%, and black clusters indicate CpG sites that were not analyzed.

We analyzed the methylation status of *DHRS3* in more detail in 100 primary tumor tissues and corresponding non-tumor tissues from 50 patients with GC by Mass-Array. We analyzed 53 CpG sites per sample (a total of 5,300 sites in 100 samples) and found that more than 71% of the CpG sites were in the amplicons (Table 1). These Mass-Array data demonstrated that the CpG sites were consistent with the BSP results (Figures 1C,D). Moreover, using Mass-Array, we detected a high degree of methylation in cancerous tissues compared with corresponding normal tissues (*P* < 0.01, Table 2). The mean methylation levels of the tumor group were increased by about 57% over the normal group (*P* < 0.01, Figure 2A). Among the CpG islands in tumors, 42% had significant changes in methylation. A two-way hierarchical cluster analysis was performed using CpGs with high discriminatory significance. For improved reliability, we included only the samples with at most one missing measure of CpG unit. Two major groups were identified: one was dominated by measurements from tumor cells and the other by normal cells (Figure 2B), although there were a few instances of crossover. Interestingly, further analysis showed that two tumor samples, which were clustered into the normal group, were actually in an early stage of tumor development (T_2_). For the 10 normal samples clustered into the tumor group, 7 were from the normal group, among which the paired tumors were in an advanced stage (T_3_ or T_4_). These results suggest that some normal tissues adjacent to the advanced tumor may have already undergone epigenetic change toward tumor formation. On the other hand, some early stage tumors may still retain the epigenetic characteristics of normal tissue.

**TABLE 1:** High-throughput methylation analysis of informative CpG sites in amplicons for DHRS3.

Gene	Amplicon size (bp)	Total no. of CpG sites in amplicon	No. of analyzed CpG sites in amplicon	No. of analyzed CpG sites per amplicons	
				Single sites	Composite sites
DHRS3	622	53	38	10	28

**TABLE 2:** Characteristics of tumor group and adjacent normal group.

	Number of patients (%)	Methylation level of tumor sample (mean ± SD)	Methylation level of normal sample (mean ± SD)	P*
Gender				
Male	39 (78%)	0.11 ± 0.11	0.07 ± 0.10	<0.001
Female	11 (22%)	0.10 ± 0.11	0.07 ± 0.08	0.005
Age (year)				
<65	36 (72%)	0.12 ± 0.12	0.07 ± 0.08	<0.001
≥65	14 (28%)	0.10 ± 0.11	0.08 ± 0.12	0.135
TNMstage				
T1, T2	13 (26%)	0.11 ± 0.10	0.09 ± 0.12	0.1
T3, T4	37 (74%)	0.10 ± 012	0.07 ± 0.09	<0.001
Vessel invasion				
Negative	41 (82%)	0.10 ± 0.11	0.08 ± 0.10	<0.001
Positive	9 (18%)	0.11 ± 0.14	0.06 ± 0.07	0.002
Lauren type				
Intestinal	33 (66%)	0.10 ± 0.10	0.07 ± 0.10	<0.001
Diffuse	7 (14%)	0.16 ± 0.22	0.09 ± 0.09	0.148
Mix type	10 (20%)	0.12 ± 0.12	0.08 ± 0.09	0.002
Differentiation				
Poor	27 (54%)	0.11 ± 0.11	0.08 ± 0.08	<0.001
Moderate	17 (34%)	0.09 ± 0.10	0.07 ± 0.13	0.048
Well	6 (12%)	0.08 ± 0.14	0.05 ± 0.06	<0.001
Total	50 (100%)	0.11 ± 0.11	0.07 ± 0.10	<0.001

*Calculated using independent samples t-test for two groups of data.

### *DHRS3* Transcription Decreased in GC Tissues and Cell Lines

**FIGURE 3:**
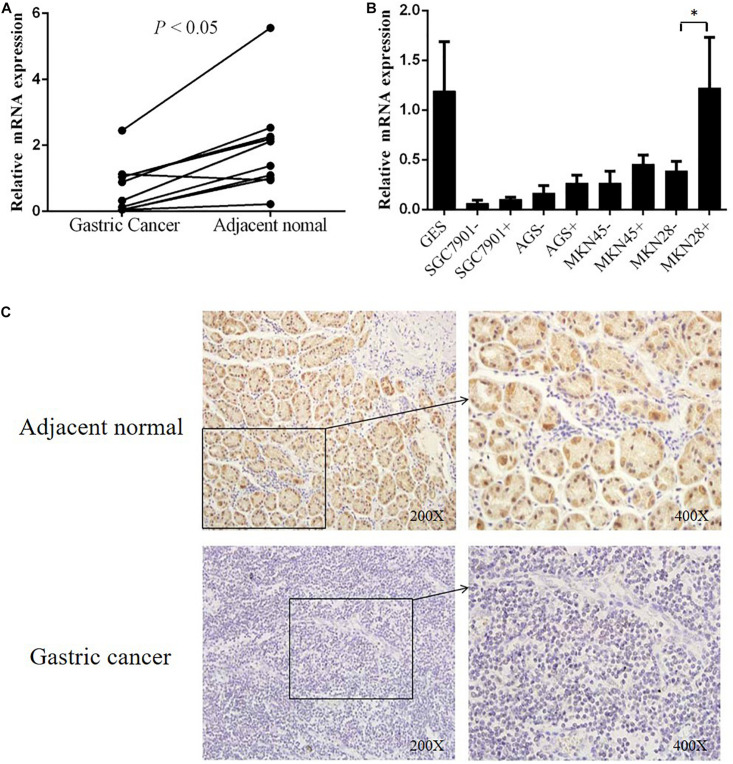
DHRS3 gene expression was significantly down regulated in primary GC tumors. **(A)** Expression levels of DHRS3 mRNA in 10 paired human gastric specimens. The *P*-value was calculated using the paired two-sample *t*-test. **(B)** mRNA expression of DHRS3 in five cell lines (GES, SGC7901, MKN45, MKN28, and AGS) before and after 5′-Aza treatment. **(C)** Representative immunohistochemical staining of DHRS3 protein of primary GC tumors and their adjacent non-tumor tissues. ^∗^*P* < 0.05.

To explore the correlation between gene transcription and promoter methylation, we next examined the mRNA levels of *DHRS3* gene in the same set of samples as above. We found that *DHRS3* mRNA levels were significantly lower in tumor tissues than in normal tissues (*P* < 0.05, Figure 3A), supporting the notion that DNA methylation of the *DHRS3* gene promoter correlates inversely with the expression of the gene. To extend this observation, we treated gastric tumor cells (SGC7901, AGS, MKN45, and MKN28) with a demethylating agent (5′-Aza) and quantified the *DHRS3* mRNA. In comparison with normal gastric GES cells, all four GC cell lines showed significantly reduced levels of mRNA. Treatment with 5′-Aza for up to 72 h resulted in increased mRNA levels in the four GC cell lines (Figure 3B). The levels of *DHRS3* mRNA were significantly increased in well-differentiated MKN28 cells treated with 5′-Aza (*P* < 0.05). In contrast, in the poorly differentiated MKN45 cells, 5′-Aza increased the mRNA level by about half that in MKN28, while the other two cell lines, AGS and SGC-7901, displayed marginal increases in *DHRS3* mRNA following 5′-Aza treatment. The response to 5′-Aza was cell-line dependent, which may also reflect differences in the initial methylation status in cultured cells.

### Downregulation of *DHRS3* Protein in Primary GC Tissues

To further support the notion that hypermethylation of the *DHRS3* gene promoter results in decreased gene expression, we next determined *DHRS3* protein levels in GC and normal tissue by immunohistochemical staining in 70 paired samples (Figure 3C). Of the normal tissues, 82.9% were strongly stained with anti-*DHRS3* antibody, while most GC samples were weakly stained (38.6%) or negative (41.4%), demonstrating that *DHRS3* protein was significantly downregulated in GC tissues compared with normal tissues (*P* < 0.01, Table 3).

**TABLE 3:** DHRS3 expression in GC tissues and matched adjacent normal tissues.

	Negative (−)	Weak (+)	Moderate (++)	Strong (+++)	*P*
Normal	2 of 70 (2.8%)	4 of 70 (5.7%)	6 of 70 (8.6%)	58 of 70 (82.9%)	<0.01
GC	29 of 70 (41.4%)	27 of 70 (38.6%)	11 of 70 (15.7%)	3 of 70 (4.3%)	

### Overexpression of *DHRS3* Inhibited Cell Proliferation and Migration of GC Cells *in vitro* and *in vivo*

**FIGURE 4:**
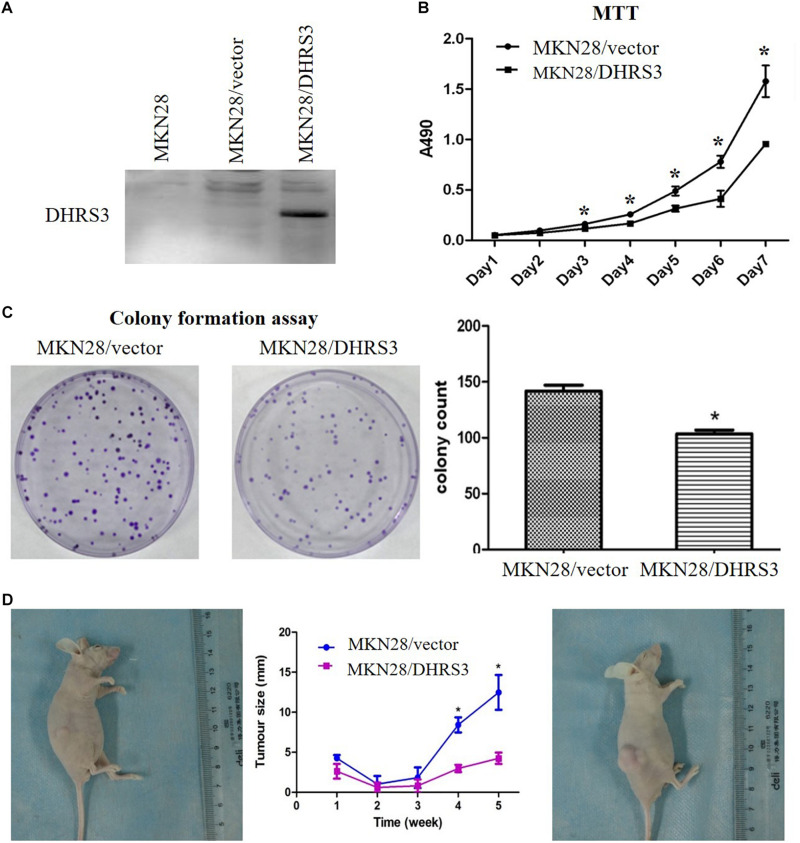
Over-expression of DHRS3 inhibited GC cell growth in cultured cells and in nude mice. **(A)** Infection of MKN28 cells with DHRS3 and control lentivirus. After selection with puromycin for 3 weeks, the expression of DHRS3 was confirmed by western blotting. **(B)** Cell growth was measured using the MTT assay. Proliferation was significantly less in MKN28/DHRS3 cells compared with MKN28/vector cells. **(C)** The colony formation assay was used to test the effect of DHRS3 on cancer cell growth. Cells were plated in 6-well plates at 300 cells/well. After 2 weeks of incubation, cells were stained. The left panel shows representative dishes after transfection with empty vector or DHRS3. Quantitative analyses of colony numbers are shown in the right panel. Values are the mean ± standard deviation (SD) of at least three individual experiments. **(D)** DHRS3 inhibited tumor growth in nude mice. A representative picture of tumor growth in nude mice subcutaneously inoculated with MKN28/DHRS3 (left) or MKN28/vector (right) cells at the end of the observation. Tumor longitudinal length (mean ± SD) was assessed every week. Tumor growth curves were plotted against days after treatment. Statistical analysis indicated significantly slower tumor growth in the MKN28/DHRS3 group. *P < 0.05.

**FIGURE 5:**
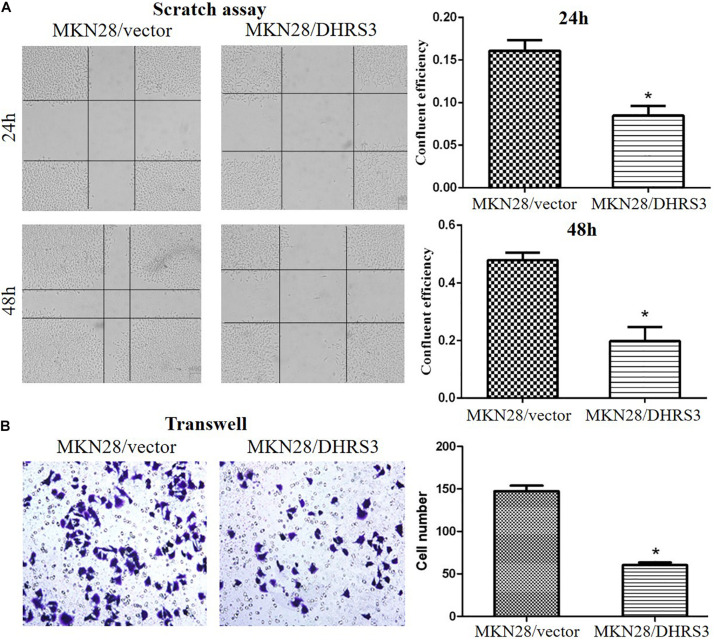
Over-expression of DHRS3 inhibited GC cell migration. **(A)** MKN28 cells were infected with DHRS3 or the empty vector. When cells were confluent, a scratch wound healing assay was performed. The scratch gap was periodically monitored and recorded. Ectopic expression of DHRS3 suppressed tumor cell migration. **(B)** DHRS3 impeded tumor cell migration in MKN28/DHRS3 cells. The results are expressed as mean ± SD of three independent experiments. *P < 0.05.

The silencing of *DHRS3* in primary GC cells suggests that *DHRS3* may function as a tumor suppressor. We infected MKN28 cells with a lentiviral expression system to enhance the expression of *DHRS3* and confirmed it by western blot analysis (Figure 4A). Ectopic expression of *DHRS3* significantly inhibited cell proliferation of MKN28 cells as determined by MTT assay (*P* < 0.05, Figure 4B). Colonies formed by cells over-expressing *DHRS3* were fewer in number and smaller in size than those formed by control cells (*P* < 0.05; Figure 4C). To further investigate the tumor suppressive effect of *DHRS3* in GC cells, we investigated whether *DHRS3* could suppress the growth of GC cells in nude mice. The mean longitudinal tumor length was assessed every week after injection with MKN28 cells infected with either *DHRS3* or vector lentivirus. The tumor growth curve showed that the mean longitudinal lengths of tumors over-expressing *DHRS3* were significantly smaller than tumors infected with vector (*P* < 0.05, Figure 4D). In addition, over-expression of *DHRS3* significantly inhibited the migration of MKN28 cells (Figures 5A,B). These results demonstrated that *DHRS3* inhibited cell growth and migration, indicating that *DHRS3* is a potential tumor suppressor, at least for GC.

### Ectopic Expression of *DHRS3* Induced G1 Phase Arrest and Apoptosis in GC Cells

**FIGURE 6:**
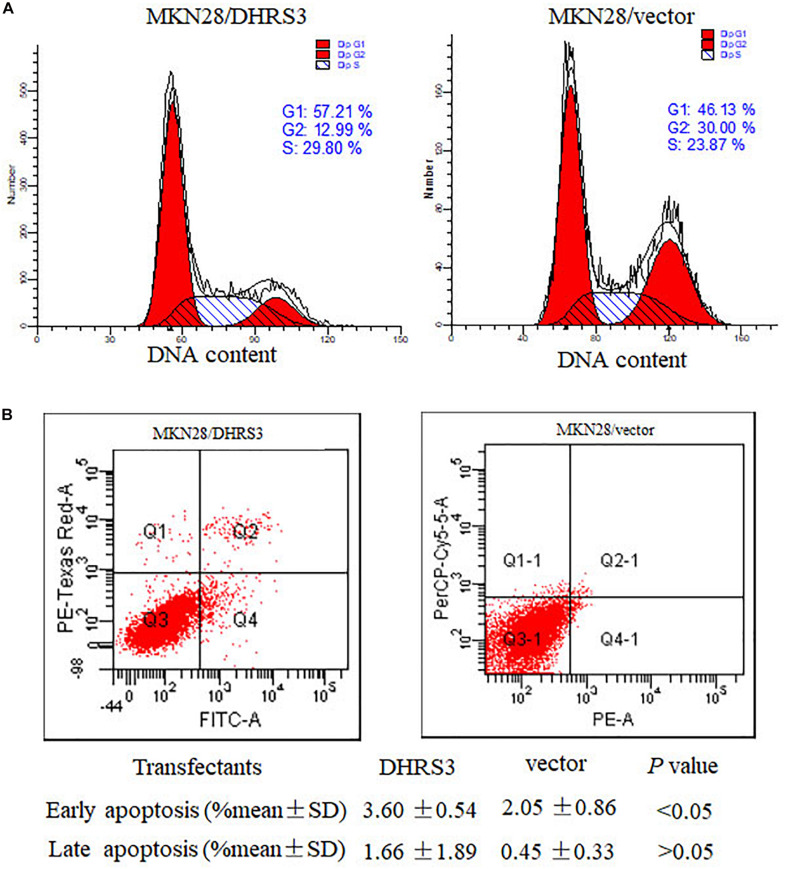
DHRS3 induced cell-cycle arrest in G1 phase and cell apoptosis. **(A)** Analysis of the cell cycle was performed by flow cytometry in the MKN28 cells stably transfected with DHRS3 or empty vector. Overexpression of DHRS3 resulted in an increase in the percentage of cells in G1 phase (57.21 vs. 46.13% in control cells). **(B)** The rate of cell apoptosis was determined by flow cytometry. Q4 indicates early apoptotic cells, Q2 shows late apoptotic cells. Results were obtained from four independent experiments.

To explore the mechanism by which *DHRS3* suppressed cell growth and proliferation, we used flow cytometry to evaluate the effects of *DHRS3* on cell cycle progression and apoptosis in MKN28 cells. Ectopic expression of *DHRS3* resulted in apparent G1 phase arrest (Figure 6A) and induced early apoptosis (*P* < 0.05, Figure 6B). These data support the inhibitory effect of *DHRS3* on cell proliferation in GC cells.

### Hypermethylation of *DHRS3* Was Associated With Histological Type and Poor Differentiation

We next extensively analyzed the correlation between the quantitative methylation based on Mass-Array data and the clinicopathological characteristics of patients including age, sex, tumor stage, histological type, differentiation, vessel invasion, and lymph node metastases (Table 4). First, we found a trend toward a positive association between hypermethylation of the *DHRS3* promoter and histological type (*P* < 0.05) and poor differentiation (*P* < 0.05). Second, because methylation of certain genes can increase with age, we analyzed the ages of patients at the time of surgery and the methylation level of the *DHRS3* gene promoter. No significant correlation was observed with regard to patient age, tumor stage, vessel invasion, or sex.

**TABLE 4:** Methylation levels in GC tissues.

	Methylation level of tumor sample (mean ± SD)	P*	Methylation level of normal sample (mean ± SD)	P*
Gender				
Male	0.11 ± 0.11	0.936	0.07 ± 0.10	0.857
Female	0.10 ± 0.11		0.07 ± 0.08	
Age (year)				
<65	0.12 ± 0.12	0.688	0.07 ± 0.08	0.109
≥65	0.10 ± 0.11		0.08 ± 0.12	
TNMstage				
T1, T2	0.11 ± 0.10	0.473	0.09 ± 0.12	0.034
T3, T4	0.10 ± 0.12		0.07 ± 0.09	
Vessel invasion				
Negative	0.10 ± 0.11	0.811	0.08 ± 0.10	0.147
Positive	0.11 ± 0.14		0.06 ± 0.07	
Lauren type				
Intestinal	0.10 ± 0.10	0.011 (For intestinal versus diffuse *P* = 0.009)**	0.07 ± 0.10	0.154
Diffuse	0.16 ± 0.22		0.09 ± 0.09	
Mix type	0.12 ± 0.12		0.08 ± 0.09	
Differentiation				
Poor	0.11 ± 0.11	0.0164 (For poor versus well *P* = 0.023)**	0.08 ± 0.08	0.073
Moderate	0.09 ± 0.10		0.07 ± 0.13	
Well	0.08 ± 0.14		0.05 ± 0.06	

*Calculated by using independent samples t-test for two groups of data and using one-way ANOVA for three groups of data. **Compared by using lsd method for two of three groups.

### Low Methylation Levels of Composite CpG 9 and CpG 10 Was Associated With Longer Survival

**FIGURE 7:**
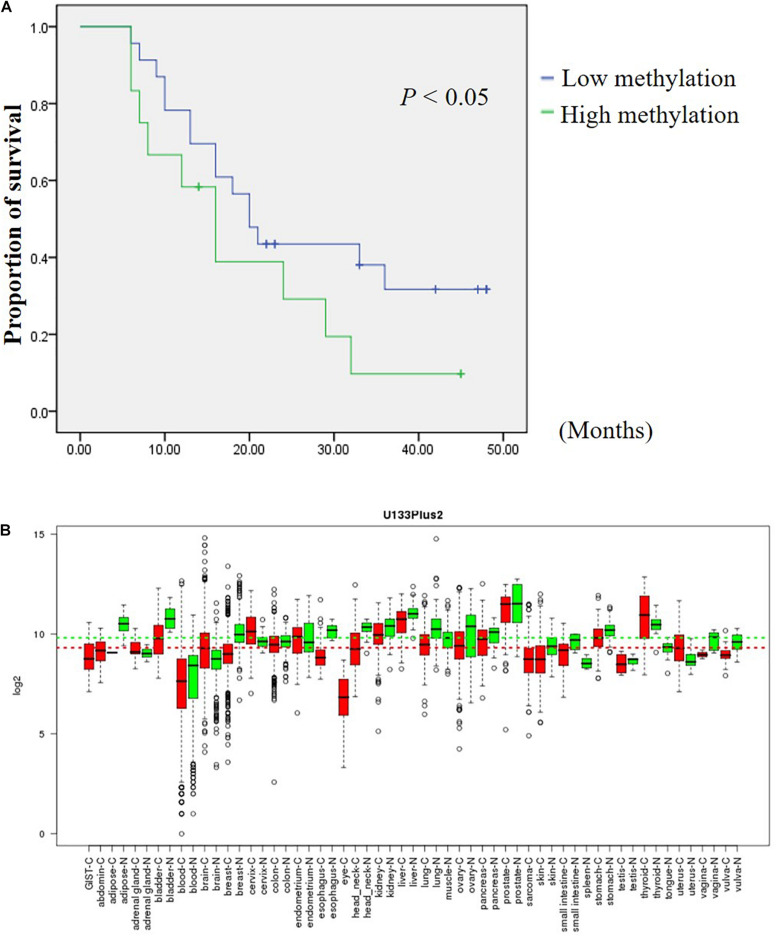
DHRS3 is down regulated in various tumors and high methylation levels of DHRS3 was associated with poor survival. **(A)** Survival curves were plotted for Kaplan-Meier survival analysis. The methylation status of CpG 9 and CpG 10 of the GC samples was used as the variable to separate two groups. Patients with high levels of methylation had poorer survival times than those with low levels of methylation (*P* < 0.05). **(B)** Expression pattern of DHRS3 mRNA in normal and tumor tissues. DHRS3 mRNA in various types of cancer was searched for in the GENT database (http://medical-genomics.kribb.re.kr/GENT/). Boxes represent the median and 25th and 75th percentiles; dots represent outliers. Red boxes represent tumor tissues; green boxes represent normal tissues. Red and green dashed lines represent the average values for tumor and normal tissues, respectively.

In an attempt to assess the prognosis of GC patients based on the methylation levels of *DHRS3* after surgery, we further analyzed the Mass-Array data and found that composite CpG sites 9 and CpG 10 were associated with an increased risk of cancer-related death. GC samples were categorized into two groups according to the methylation level of CpG 9 and CpG 10 in the GC samples. Among them, 65.7% had below average methylation levels and 34.3% of GC samples showed above average methylation of CpG 9 and CpG 10. Kaplan–Meier survival curves demonstrated that GC patients with low methylation levels of CpG 9 and CpG 10 had significantly longer survival times (median, 20 months) than those with high methylation levels (median, 16 months; *P* < 0.05; Figure 7A).

## Discussion

Tumorigenesis is a multistep process with gradual accumulation of genetic and epigenetic alterations. An increasing number of tumor suppressor genes have been found to harbor CpG islands in their gene promoters and the first exon where the epigenetic regulation predominately occurs, indicating an alternative mechanism to genetic inactivation of tumor suppressor genes. In this study, we found that the *DHRS3* gene was hypermethylated in GC patients and correlated with decreased *DHRS3* mRNA and protein levels in primary tumors. Demethylation by treatment with 5′-Aza increased the expression of *DHRS3* in cultured GC cells. Moreover, high degree of *DHRS3* methylation was associated with unfavorable clinicopathological features and shorter survival times in GC patients. These results proved that *DHRS3* downregulation may play an important role in the development of GC and may carry tumor suppressor potential.

As a member of the SDR family, *DHRS3*, encodes an enzyme involved in the metabolism of retinol (vitamin A). Retinol, apart from its unique role in the production of visual pigments, also has potential preventive effects on malignant neoplasms (Morriss-Kay and Ward, 1999; Chandrasekaran et al., 2000). It has been noted that vitamin A may influence gastric carcinogenesis (Larsson et al., 2005; Wang, 2005) and that it is inversely associated with GC (Stahelin et al., 1984; Stehr et al., 1985; De Stefani et al., 2000). *DHRS3*, which is frequently deleted or rearranged in neuroblastomas, is critical for the generation of a storage form of retinol. Further study has elucidated that deletion of *DHRS3* might contribute to cancer development and progression by reducing the production of vitamin A (Cerignoli et al., 2002). Thus, exploring the biofunction of *DHRS3* in GC will help to enable an understanding of the mechanisms underlying GC.

The GENT database indicates that *DHRS3* mRNA is down-regulated in cancers of the bladder, lung, ovary, skin, stomach, and vagina compared with corresponding normal tissues, but is upregulated in cancers of the brain, cervix, and uterus (Figure 7B). The distinct expression status of *DHRS3* among different tumor types needs further investigation to gain an understanding of the physiological function of the gene during tumorigenesis. We demonstrated that the protein expression of *DHRS3* was downregulated in a large percentage of gastric tumor specimens compared with corresponding non-tumor tissues. This dynamic change in the expression of *DHRS3* between primary tumors and corresponding normal samples implied that *DHRS3* may have important effects in the development and progression of GC.

To further study the supposed tumor suppressor function of *DHRS3* in GC, we performed both *in vitro* and *in vivo* assays. Re-expression of *DHRS3* significantly inhibited both growth and clone formation of cultured MKN28 cells and reduced tumor size in nude mice. Furthermore, ectopic expression of *DHRS3* induced G1 phase arrest and apoptosis. Taken together, it is implied that *DHRS3* plays the role of a tumor suppressor in GC.

Hypermethylation of CpG islands in gene promoter regions is an important mechanism of gene inactivation during tumorigenesis. By using BSP and Mass-Array, we found that the promoter region of *DHRS3* gene was frequently methylated in GC patients. Furthermore, our findings showed that some CpG sites are more frequently methylated in the promoter region. This result suggested that these candidate sites may play a more important role in *DHRS3* transcription, and this finding was consistent with those from a recent study showing that not all CpG sites in the promoter region have equal function (Zou et al., 2006).

Although promoter methylation has been widely studied in GC patients, most reports lack quantitative analysis. The Mass-Array system allows robust analysis of the methylation status of primary tumors compared with normal tissues for further use in molecular detection. In this study, we analyzed the majority of the cases by Mass-Array and found that *DHRS3* promoter methylation was tumor specific (*P* < 0.01). Tissues located 3–5 cm away from the tumor are currently considered adjacent normal tissues. However, in our two-way hierarchical cluster analysis, 10 morphologically normal samples clustered into the tumor group. For 7 of these 10 samples, their paired tumor samples belonged to T_3_ or T_4_ stage. This result suggested that the morphologically normal tissues may have already undergone epigenetic change, and this may provide a new insight for resection standards in the future. Most importantly, we found that high levels of *DHRS3* methylation correlated significantly with shorter survival time in GC patients. Thus, the inactivation of *DHRS3* by methylation of the promoter would favor tumor progression and lead to a worse outcome for patients. To our knowledge, this is the first report to demonstrate that *DHRS3* functions as a tumor suppressor gene in GC patients. *DHRS3* promoter hypermethylation in GC patients can be used as an epigenetic biomarker not only for making a diagnosis but also for predicting the prognosis.

In conclusion, we identified *DHRS3* as a specific target for aberrant CpG island hypermethylation in GC patients and its inactivation may contribute to the malignant behavior of GC cells. These findings provide a basis for further investigation into *DHRS3* as a tumor-suppressor gene and the role of its inactivation in the pathogenic development of GC.

## Data Availability Statement

The raw data supporting the conclusions of this article will be made available by the authors, without undue reservation.

## Ethics Statement

The studies involving human participants were reviewed and approved by Xijing Hospital of the Air Force Medical University. The patients/participants provided their written informed consent to participate in this study. The animal study was reviewed and approved by Xijing Hospital of the Air Force Medical University.

## Author Contributions

SS, KX, and XB: molecular biology experiment. XB, SS, TY, and CF: article writing. WK, ZQ, and NY: experimental design. BB and SX: animal experiment. XB and SX: epigenetics experiment. All authors contributed to the article and approved the submitted version.

## Conflict of Interest

The authors declare that the research was conducted in the absence of any commercial or financial relationships that could be construed as a potential conflict of interest.

## Footnotes

1 http://www.urogene.org/methprimer/index1.html
